# Allocation of Carbon from an Arbuscular Mycorrhizal Fungus, *Gigaspora margarita*, to Its Gram-Negative and Positive Endobacteria Revealed by High-Resolution Secondary Ion Mass Spectrometry

**DOI:** 10.3390/microorganisms9122597

**Published:** 2021-12-16

**Authors:** Yukari Kuga, Ting-Di Wu, Naoya Sakamoto, Chie Katsuyama, Hisayoshi Yurimoto

**Affiliations:** 1 Graduate School of Integrated Sciences for Life, Hiroshima University, 1-7-1 Kagamiyama, Higashi-Hiroshima 739-8521, Hiroshima, Japan; ckatsu@hiroshima-u.ac.jp; 2 Institut Curie, Université PSL, CNRS UMS2016, Inserm US43, Université Paris-Saclay, Multimodal Imaging Center, 91400 Orsay, France; Ting-di.wu@curie.fr; 3 Isotope Imaging Laboratory, Creative Research Institution, Hokkaido University, Kita-21 Nishi-11, Kita-ku, Sapporo 001-0021, Hokkaido, Japan; naoya@ep.sci.hokudai.ac.jp; 4 Department of Natural History Sciences, Hokkaido University, Kita-21 Nishi-11, Kita-ku, Sapporo 001-0021, Hokkaido, Japan; yuri@ep.sci.hokudai.ac.jp

**Keywords:** arbuscular mycorrhiza, endobacteria, stable isotope labelling, secondary ion mass spectrometry

## Abstract

Arbuscular mycorrhizal fungi are obligate symbionts of land plants; furthermore, some of the species harbor endobacteria. Although the molecular approach increased our knowledge of the diversity and origin of the endosymbiosis and its metabolic possibilities, experiments to address the functions of the fungal host have been limited. In this study, a C flow of the fungus to the bacteria was investigated. Onion seedlings colonized with *Gigaspora margarita*, possessing *Candidatus* Glomeribacter gigasporarum (*Ca*Gg, Gram-negative, resides in vacuole) and *Candidatus* Moeniiplasma glomeromycotorum (*Ca*Mg, Gram-positive, resides in the cytoplasm,) were labelled with ^13^CO_2_. The ^13^C localization within the mycorrhiza was analyzed using high-resolution secondary ion mass spectrometry (SIMS). Correlative TEM-SIMS analysis of the fungal cells revealed that the ^13^C/^12^C ratio of *Ca*Gg was the lowest among *Ca*Mg and mitochondria and was the highest in the cytoplasm. By contrast, the plant cells, mitochondria, plastids, and fungal cytoplasm, which are contributors to the host, showed significantly higher ^13^C enrichment than the host cytoplasm. The C allocation patterns implied that *Ca*Mg has a greater impact than *Ca*Gg on *G. margarita*, but both seemed to be less burdensome to the host fungus in terms of C cost.

## 1. Introduction

The arbuscular mycorrhizal fungi, Glomeromycotina, are obligate mutualistic symbionts of plants that colonize the roots. The fungal–bacterial interaction is well known in this subphylum and the presence of Gram-positive bacteria (GPB) within the fungal cell has been widely observed. Furthermore, some members of Diversisporales also possess Gram-negative bacteria (GNB) [1,2]. The taxonomic position of GNB was first recognized as beta-proteobacteria, *Burkholderia* related (BRE), and later named *Candidatus* Glomeribacter gigasporarum (*Ca*Gg) [3]; the closest group was *Mycoavidus*, which was known as an endosymbiont of *Mortierella elongata* [4,5]. GPB, a sister clade of Mycoplasmatales and Entomoplasmatales, has been referred to as *Mycoplasma* related endobacteria (Mre) [6] and named *Candidatus* Moeniiplasma glomeromycotorum (*Ca*Mg) [7]. Ultrastructural observations have shown distinct niches in the host cells between *Ca*Gg and *Ca*Mg: the former resides in the vacuole and the latter in the cytoplasm [2]. Since their presence was discovered [8], their obligate lifestyle has been suspected from unsuccessful attempts to culture them, and recent genomic analysis has confirmed that *Ca*Mg and *Ca*Gg have reduced genome sizes and lack the ability to biosynthesize many amino acids, and that the former also lack nucleic acids [9,10]. These obligate dependencies agree with the proposed propagation manner of vertical transmission [11], which is estimated to have originated as long as 400 million years ago [12].

In the plant–fungal symbiosis of arbuscular mycorrhiza (AM), the fungus obtains carbon (C) from the host plant; in return, the host obtains mineral nutrients, such as phosphorus (P), and nitrogen (N), from the partner. However, regarding the fungus–endobacteria symbiosis in Glomeromycotina, the relationship and functions are still poorly understood. In other fungus–endobacteria symbioses, *Burkholderia rhizoxinica* is responsible for the pathogenicity of the fungal host, *Rhizopus microsporus*, causing rice seedling blight [13,14]. In *Mycoavidus cysteinexigens* and *Mortierella elongata* symbiosis, the presence of the bacteria influences the host fungus, resulting in the reduction of the host fungal growth rate by comparison with the fungus when isolated from the endobacteria [5]. In the Glomeromycotina endobacteria, *Gigaspora margarita* proliferated after removing its only symbiont *Ca*Gg and the relationship has been proposed to be facultative for the fungal host [15]. Moreover, recent approaches have shown the bacterial influence on the host fungus by comparing two lines with and without bacteria using transcriptomics and proteomics and proposed that the bacterial presence improves the fungal ecological fitness [16].

Secondary ion mass spectrometry (SIMS) employs an energetic primary ion beam to bombard the sample surface to trigger the ejection of secondary ions. These secondary ions can be identified by their mass through a mass spectrometer and their intensities can be measured individually. The imaging SIMS has been recognized for its high potential in biological applications, mainly because the technique covers all elements from hydrogen [17]. Modern, dynamic SIMS instruments offer high-mass resolution along with high lateral resolutions, allowing us to use them for investigating transfers of biological elements within and between organisms, and between organisms and the environment by tracing stable isotopes at an ultrastructural level [18,19,20]. The purpose of this study is to understand C allocation from the host fungus to its endobacteria. To achieve this aim, we applied ^13^C labelled CO_2_ to the arbuscular mycorrhizal plant colonized with the *G. margarita* C strain (MAFF520054) known to harbor both *Ca*Gg and *Ca*Mg [2,7] and analyzed the flow in the quadruplet symbiosis, mainly focusing on transfer from the fungus to the two bacteria. The localization of the isotope enrichment was imaged by high-resolution SIMS, further enhanced by the correlative transmission electron microscopy (TEM)—nanoSIMS approach, similar to that for elemental distribution mapping [21].

## 2. Materials and Methods

An onion seedling was grown in a root box system (Figure S1). The wall of the bottom of a plastic Petri dish was removed in one part. The soil mixture (river sand:field soil:horticultural soil = 5:4:1) was filled in the dish and the surface was covered with a non-woven-fabric, and a cellulose acetate membrane (47 mm in diam., 0.8 μm pore size; ADVANTEC, Tokyo, Japan) was placed on the top so that the developed roots were separated from the soil. An onion seedling was put on the filter and 30 spores of *G. margarita* Becker & Hall (MAFF520054, Ministry of Agriculture, Forestry and Fisheries, Genebank, Tsukuba, Japan) were inoculated beside the roots. The roots were covered by the lid where the sidewall was partially removed, and the root box was covered with aluminum foil and cultured in a growth room while being kept in a vertical position for 8 weeks (25 °C, 16 h light–8 h dark). The mycorrhizal plants forming new spores were enclosed in a plastic bag (4.5 L), and 225 mL of ^13^CO_2_ (^13^C 99%, ISOTEC Inc. Miamisburg, OH, USA) was injected into the bag with a syringe. The labelling was carried out as described above. After labelling for 48 h, the plant roots were fixed in a mixture of freshly prepared 2% paraformaldehyde and 2.5% glutaraldehyde in 50 mM PIPES buffer (piperazine-1,4-bis (2-ethanesulfonic acid), pH 6.9) for two hours, rinsed in the buffer, and the colonized areas were then excised under a binocular microscope. The root pieces were further fixed by 1% OsO_4_ in the 50 mM PIPES buffer for two hours. Then, the sample was rinsed with distilled water, dehydrated with a graded ethanol series (50, 70, 80, 90, 95, and 100%), infiltrated, embedded in an epoxy resin (Embed-It^TM^; Polysciences Inc., Warrington, PA, USA), and polymerized at 70 °C.

Semi-thin sections (200–500 µm thickness) of the resin-embedded samples were obtained using an ultramicrotome (Leica EM UC7, Leica microsystems, Wetzlar, Germany) and stained with Toluidine blue O (0.05% in 1% sodium borate in H_2_O), and samples showing colonized areas were selected using a light microscope (Axio Imager A1, Carl Zeiss, Jena, Germany). For observation, we used an isotope microscope system at Hokkaido University (ims-1270 (CAMECA, Paris, France) coupled with a solid-state ion imager (SCAPS)). Sections that were 1 µm thick were placed on a drop of filtrated water on a 7 mm × 7 mm silicon wafer and adhered by heating of a hot plate. In this system, isotope images were sequentially obtained using a Cs^+^ primary ion in the order ^32^S^−^, ^13^C^−^, ^12^C^−^, ^13^C^−^, ^31^P^−^, ^16^O^−^, and ^12^C^14^N^−^ [18,22]. Two ^13^C images were averaged, then the calculated image was divided by the image of ^12^C. The result was a ^13^C/^12^C ratio image that was multiplied by 100 (r13C). The percentage of the natural abundance of ^13^C is around 1%; therefore, a value of more than 1 for r13C indicates the presence of the isotope tracer.

For correlative TEM-NanoSIMS analysis, sections that were 120 nm thick were obtained by an ultramicrotome using a diamond knife, placed on a formvar carbon coated copper grid (200 mesh, EM Japan Co. Ltd., Tokyo, Japan), and the ultrastructure of the roots was recorded (JEM-1400, JEOL Ltd., Tokyo, Japan). Then, the grid was analyzed by NanoSIMS-50 (CAMECA, Paris, France) where ^12^C^−^, ^13^C^−^, ^12^C^14^N^−^, ^31^P^−^ images were taken simultaneously using a Cs^+^ primary ion, and an r13C image was produced by dividing the ^13^C image by the of ^12^C image and multiplying the result by 100. Region of interest (ROI) analysis was conducted on the fungal cytoplasm, vacuoles, mitochondria, Gram-negative, and Gram-positive bacteria, as well as on plant cytoplasm, vacuole, mitochondria and proplastids using ImageJ (Fiji). The ROIs within one image were averaged, and the averaged values were used for a multiple comparison analysis (replicates are the number of images that contain the structures; Tukey–Kramer method; Statcel-the Useful Add-in Forms on Excel-4th ed., OMS Publishing Inc., Tokyo, Japan).

## 3. Results

**Figure 1:**
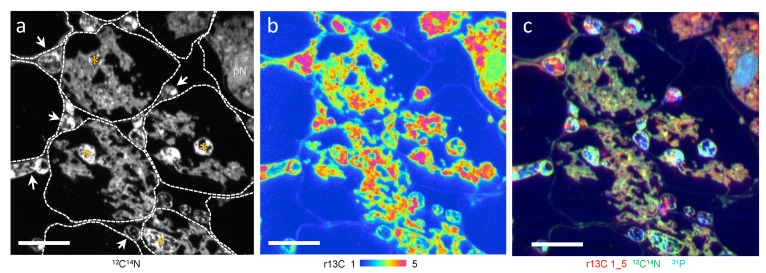
A part of ^13^CO_2_ labelled *Allium cepa* mycorrhizal root section colonized with *Gigaspora margarita* (ims-1270 equipped with SCAPS). Cells showing intercellular hyphae (arrowheads) and arbuscules composed of trunk hyphae (*) and fine branches. Fine branches are surrounded by plant cytoplasm. (**a**) ^12^C^14^N image. Boundaries of plant cells are shown by dotted lines. (**b**) ^13^C/^12^C × 100 (r13C). (**c**) Overlay images of r13C (red), ^12^C^14^N (green), and ^31^P (blue). pN, plant nucleus. Bars: 20 μm.

In the onion roots, *G. margarita* form Arum-type mycorrhiza where intercellular hyphae grow between the host cell and form an arbuscule within cortical cells (Figure 1). Arbuscule-forming hyphae, including a thick trunk and fine branches, were surrounded by the peri-fungal membrane derived from the host plasma membrane. An r13C higher than natural abundance was observed in the mycorrhizal root, especially associated with the host and arbuscule complex and intercellular hyphae (Figure 1a–c). An overlay image of r13C (red), ^12^C^14^N (green), and ^31^P (blue) showed the plant nucleus as light blue (Figure 1c).

**Figure 2:**
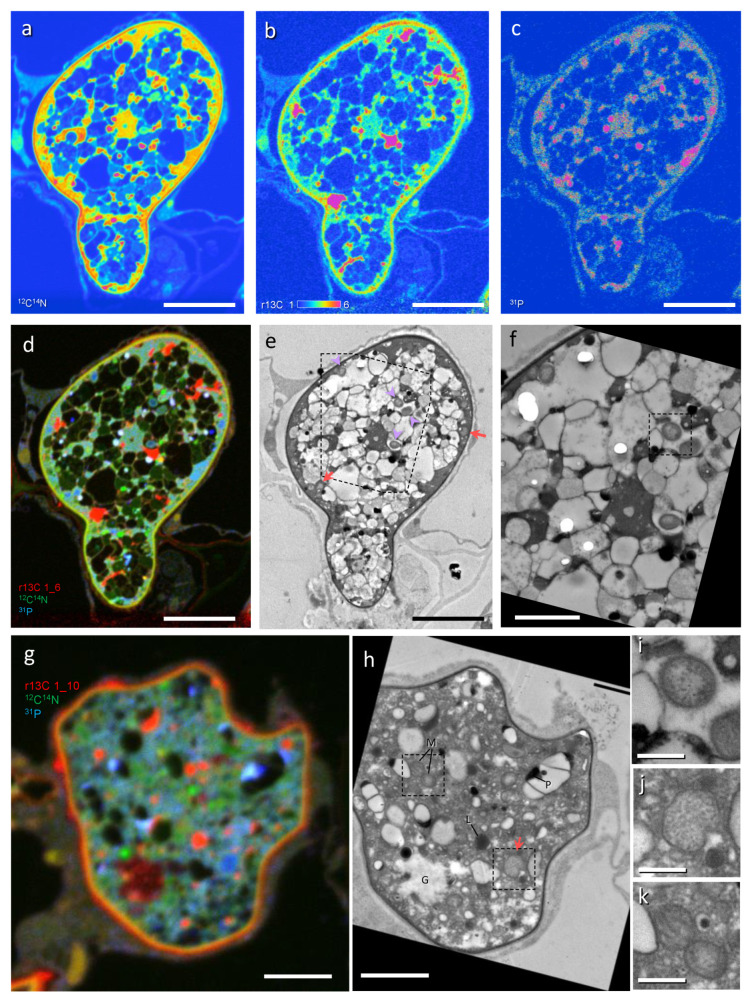
Correlative imaging by transmission electron microscopy (TEM) and NanoSIMS-50 of a ^13^CO_2_ labelled *Allium cepa* root colonized with *Gigaspora margarita*. (**a**) ^12^C^14^N. (**b**) ^13^C/^12^C × 100 (r13C). (**c**) ^31^P. (**d**,**g**) Overlay images of r13C (red), ^12^C^14^N (green), and ^31^P (blue). (**e**,**f**,**h**–**k**) Corresponding TEM of (**a**–**d**,**g**). G, glycogen; M, mitochondrion; L, lipid body; P, polyphosphate. Arrows, *Candidatus* Moeniiplasma glomeromycotorum; arrowheads, *Candidatus* Glomeribacter gigasporarum. Dashed squares in (**e**,**f**,**h**) show areas corresponding to (**i**,**j**,**k**), respectively. Bars: (**a**–**e**), 5 μm; (**f**–**h**), 2 μm; (**i**–**k**), 0.5 μm.

In correlative TEM-NanoSIMS-50 (Figure 2), eleven *Ca*Mg and six *Ca*Gg were identified structurally in trunks and intercellular hyphae from seven images of SIMS (Table S1). The *Ca*Mg was coccoid, often constricted in the center (Figure 2j), 0.44 µm (0.34–0.55 µm) in width (measured in ten cells), and present in fungal cytoplasm (Figure 2e,f,h,j). The *Ca*Gg was rod-shaped, 0.45 µm (0.41–0.51 µm) in width (measured in six cells), and present in the vacuoles (Figure 2e,f,i). Overlay images of r13C (red), ^12^C^14^N (green), and ^31^P (blue) showed that *Ca*Mg was light blue and the *Ca*Gg was greenish blue, indicating that both contain P in high amounts (Figure 2d,g). Although only three hyphal profiles include both bacteria, *Ca*Mg contains more ^31^P than *Ca*Gg (^31^P ratios of *Ca*Mg/*Ca*Gg ranged between 2.4 and 2.7, Table S1). The bacterial blue colors distinguished them from fungal mitochondria (Figure 2d,g,k) that are clear green (less ^31^P) and often accompanied by r13C elevation outside, seen as an orange fringe. Fungal vacuoles contained ^31^P-rich and electron dense granules of polyphosphate, which were deposited during sample preparation. Together with the morphology, the blue color in the overlay image and high electron density differentiate it from *Ca*Gg.

**Figure 3:**
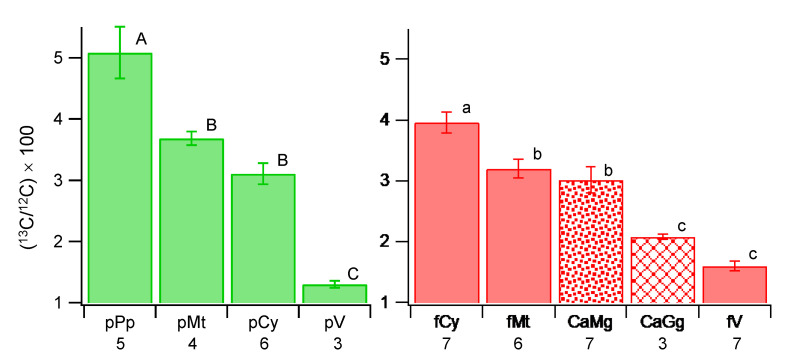
Multiple comparison of ^13^C ratios of plant structures (**left**) and fungal structures and endobacteria (**right**). CaGg, *Candidatus* Glomeribacter gigasporarum; CaMg, *Candidatus* Moeniiplasma glomeromycotorum; fCy, fungal cytoplasm; fMt, fungal mitochondrion; fV, fungal vacuole; pCy, plant cytoplasm; pMt, plant mitochondrion; pPp, plant plastid; pV, plant vacuole. The averages of the structures were obtained in each image, which was used for the multiple comparison tests (Tukey–Kramer method), which were conducted in the plant and fungal structures separately. Different letters show statistically significant differences among structures (plant, A–C; fungal and bacteria, a–c) based on the Tukey–Kramer method at *p* < 0.05. Bar, standard error of the mean (N = number of images analyzed in the structure—shown under each structure).

All structures analyzed showed a higher ^13^C ratio than the natural abundance; therefore, ^13^CO_2_ fixed by the host plant was transferred to the fungus, and further to the endobacteria (Figure 3 and Figure S2, Tables S1 and S2). The r13C of *Ca*Mg was 3.02 ± 0.58 (average ± standard deviation) and *Ca*Gg was 2.08 ± 0.08, which were statistically significant (*p* < 0.05). The values of both bacteria were statistically lower than fungal cytoplasm (3.96 ± 0.45, *p* < 0.01), although *Ca*Mg had direct contact with it. Fungal mitochondrion (3.20 ± 0.38) was slightly higher than *Ca*Mg, but significantly lower than that of the fungal cytoplasm. By contrast, in plant cells, plastids (5.09 ± 0.94) and mitochondria (3.69 ± 0.22) showed higher r13C than the plant cytoplasm (3.11 ± 0.42). The fungal cytoplasm was higher than the plant cytoplasm, which was statistically significant when all the ROIs of all the structures were analyzed (Figure S3), indicating a higher uptake of C molecules by the fungus from the host. In the fungal cytoplasm, lipid and glycogen formation proceeded.

## 4. Discussion

This study revealed for the first time the C flow within a quadruplet symbiosis, from the plant through the arbuscular mycorrhizal fungus and to the two fungal endobacteria.

The *G. margarita* C strain used in this study has been pot-cultured in our laboratory for two decades and the presence of *Ca*Gg and *Ca*Mg was confirmed based on the 16S rRNA gene sequences (unpublished data). Their niches in the host cell and the structures agreed with the previous studies [6,15]; rod-shaped bacteria resided in the vacuoles assumed as *Ca*Gg, and coccoid bacteria residing in the cytoplasm as *Ca*Mg. Because of the size, less than 0.5 µm in width, an ultrastructural resolution was essential to find the endobacteria, and to distinguish *Ca*Mg from mitochondria and other vesicular structures and *Ca*Gg from granularly deposited polyphosphate during ethanol dehydration [23,24]. The biomasses of the bacteria were unknown, but the *Ca*Mg was found more often than the *Ca*Gg in this study, which agreed with the study where the quantities of the two bacteria were analyzed using real-time qPCR [2].

In the biological application of SIMS, several requirements and concerns exist: analysis under a high vacuum condition, the necessity of a flat surface, sample erosion due to the destructive character of the SIMS technique, and the deposition of reactive primary ions as Cs^+^ at a high dose on the surface by measurement. Because of the former two requirements, biological samples prepared for electron microscopy are compatible. For the latter two reasons, especially in the correlative TEM-SIMS, the structural data by TEM must be taken prior to NanoSIMS analysis. The thickness of the section must be thin enough for the transmission of electrons and sufficiently thick (robust) to stand for SIMS measurement. Regarding labelling, because this method measures the substances, strengthened conditions seem more necessary than the method that uses radioactive tracers. The two preliminary experiments using a lower quantity of label and shorter labelling time could not allow the detection of ^13^C other than the natural abundance in mycorrhizal roots.

The isotope ratio permits the trafficking of isotopes. A higher ratio implies the deposition of newly fixed ^13^C in the structure unless there is a mechanism of only ^12^C being removed with the same amount. The elevated ratio is most likely accompanied by an increase in the total amount of ^13^C, but it does not necessarily mean that double the value is double the amount. The replacement of ^12^C with ^13^C can also bring a higher ratio without changes in the total quantity. A low ^13^C ratio can be attributed to no, less, or slow allocation and a situation where molecules are metabolized and removed quickly, such as oxygenic respiration in mitochondria. This study showed the ratio value in the plant mitochondria was always slightly higher than in its cytoplasm, even though the difference was not significant, which may indicate a footprint of metabolizing ^13^C in the mitochondria. This study revealed differences in C allocation between *Ca*Mg and *Ca*Gg and was higher in *Ca*Mg. Additionally, compared to the *Ca*Mg, C allocation was slightly higher in the fungal mitochondria and was significantly higher in the fungal cytoplasm. This order was consistent with the observations found in other independent experiments (unpublished data). The *Ca*Gg lives in the vacuole, which exhibits the lowest r13C, indicating active transport of labelled ^13^C molecules by *Ca*Gg from the surrounding area. Fungal vacuoles store polyphosphate in a dispersive form [23,24,25,26] and amino acids [27]. In AMF, arginine has been proposed as a candidate for storage in the vacuoles [28]. Additionally, *Ca*Gg lacks the ability to biosynthesize amino acids, such as arginine [1,9], suggesting that it is being supplied from the surrounding area. In this study, a vacant type of vacuole was common in all images and analyzed, where ^12^C^14^N was low but the methodological artifact should be taken into account. Indeed, the hypha of Figure 2a–f (Table S1, Image #4) shows two types of vacuoles; one is vacant and the other was filled by N in a dispersed form. The vacuoles containing *Ca*Gg showed that the r13C of the vacuole area was lower than *Ca*Gg but was not significant and the ^12^C^14^N was the same as that of the N-rich vacuoles (Figure S4). This case implied a possibility of *Ca*Gg influence in the residing vacuole, but more data with different sample preparations are necessary.

The *Ca*Mg and the fungal mitochondria, showed lower r13C than the fungal cytoplasm. By contrast, the fungus, a mutualistic partner of the host plant, was awarded ^13^C, as the ^13^C ratio of the cytoplasm was higher than the host cytoplasm. This is also the case of the plant plastids and mitochondria, accumulating more tracers than the plant cytoplasm. Progress in understanding fungal genomes has suggested that the lack of ability of de novo synthesis of 16C fatty acid seems to be a common characteristic of AMF [29,30,31]. The fungi are dependent on carbon from the host plants, and the intraradical hyphae are the site of C assimilation. In this study, the multiple comparison of r13C using all structures showed, indeed, that fungal cytoplasm was the second highest after plastids and higher than plant cytoplasm (Figure S3). ROI setting of the fungal cytoplasm was difficult because the vacuoles and lipid bodies occupied most of the cell volume and the active lipid and glycogen syntheses in the cytoplasm made the distribution of the isotope heterogenous. Therefore, to discuss the level of r13C by comparing it with the fungal cytoplasm or the surrounding environment may not be adequate. The *Ca*Mg had the same level of r13C as the fungal mitochondria and plant cytoplasm, suggesting the *Ca*Mg has a higher cost than *Ca*Gg for its fungal host in terms of carbons. Indeed, it has been suggested that *Ca*Mg obtains the molecules from the fungal host, based on the missing amino acids and nucleic acid synthesis abilities [10]. Particularly, the way to exist in the host cytoplasm without being encased by a host membrane also suggests easy access to the host molecules.

Endobacterial functions in the host remain a challenging topic. Both *Ca*Gg and *Ca*Mg are obligate symbionts of the host fungus, and AMF are also obligate symbionts of plants. In the host plant cells, mitochondria and plastids are located near the fungal hyphae with higher ^13^C ratios, strongly indicating their significant roles of the C transfer to the fungus. This study showed higher ^13^C ratios of plant organelles, mitochondria and plastids, which are evolved by endosymbiosis and are perpetual residents, compared to that of plant cytoplasm. The higher allocation of C revealed by the higher ^13^C ratio in the cytoplasm was also shown in the mutualistic fungal partner. Applying this to the endobacterial patterns, the *Ca*Gg and *Ca*Mg seem to be less burdensome to the host fungus in terms of the C cost. They seem not to be mutualists but also not parasitic partners bringing considerable loss or damage to the host. This view may explain the long resident history [5,6]. The influence of the host measured by the C allocation seems to be larger in *Ca*Mg than *Ca*Gg, which might indicate a stronger dependency caused by the significantly reduced genome size in *Ca*Mg [9,10]. Although the *Ca*Gg-free strain did not change the performances as AMF [15], recent studies have shown differences in transcriptional activity by the presence and absence of *Ca*Gg [32], and the influence of *Ca*Gg on the fungus, such as a modulation of fungal protein expression, fatty profiles, fungal growth, oxidative stress, and respiration [16,33]. In this study, allocations of ^13^C in the endobacteria were lower than the surrounding environments. The ^13^CO_2_ labelling conditions used in this study were found after two experiments ended with no labelling of mycorrhizal plants and the fungus. If the bacterial life cycles take several days, longer incubation with ^13^CO_2_ may be necessary. The low C to N ratio of prokaryotic cells and the stable nutrient-rich environments may explain the low or slow C allocation found in the endobacteria, or the humble allocation may show the control of the partners by the host. The methodology, tracing stable isotopes in the whole organism level and imaging the ratios at the organelle level in situ, opened a new strategy to studying intact cell and symbiotic functions. The results of this study clearly demonstrated the carbon flow hypothesized by Ghignone et al. (2012) and revealed a low carbon cost of the fungal endobacteria. N transfer, the bacterial life cycle, and their interactions with fungal hosts in Glomeromycotina endobacteria symbioses require further investigation.

## Supplementary Materials

The following are available online at https://www.mdpi.com/article/10.3390/microorganisms9122597/s1: Figure S1, Root box culture and labelling, Figure S2, ROIs of Figure 2, Table S1, Averages of r13C, 31P, and 12C14N of ROIs of seven images analyzed, Table S2. r13C of structures and the multiple comparison within plant and fungus (Tukey-Kramer method), Figure S3, Multiple comparisons of r13C of all structures (all ROIs of Gram-negative and positive bacteria, and fungal and plant organelles), Figure S4, ROI analysis of fungal structures of #4 (Figure S1a–f).
