# Supplementary material for: Allocation of Carbon from an Arbuscular Mycorrhizal Fungus, Gigaspora margarita, to Its Gram-Negative and Positive Endobacteria Revealed by High-Resolution Secondary Ion Mass Spectrometry
Source: Microorganisms. 2021 Dec 16;9(12):2597. doi: 10.3390/microorganisms9122597 (PMC8705746; doi:10.3390/microorganisms9122597)
Supplement: Supplementary file 1 [file microorganisms-09-02597-s001.zip › microorganisms-1485502-supplementary.pdf]

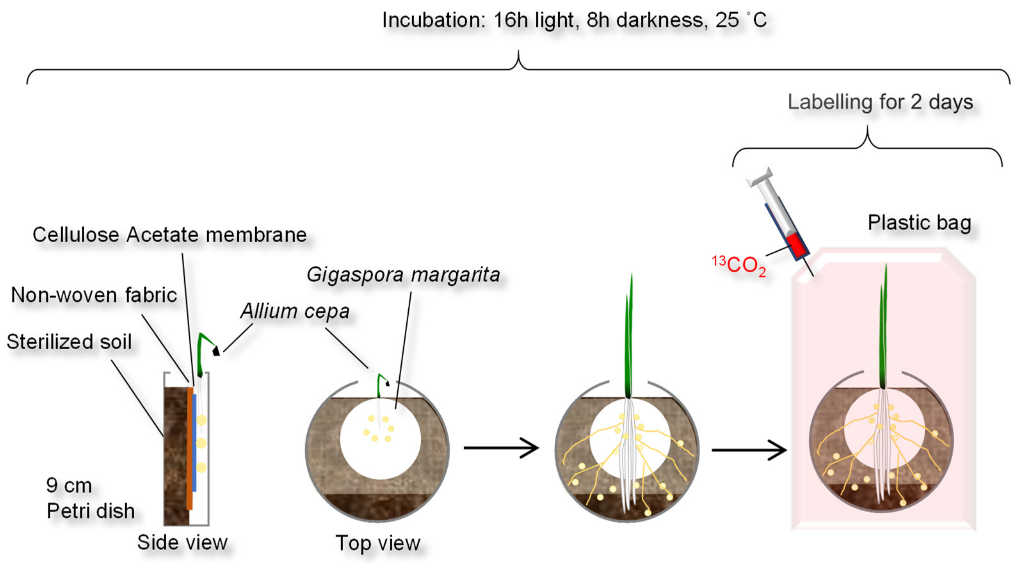

Figure S1 Root box culture and labelling.

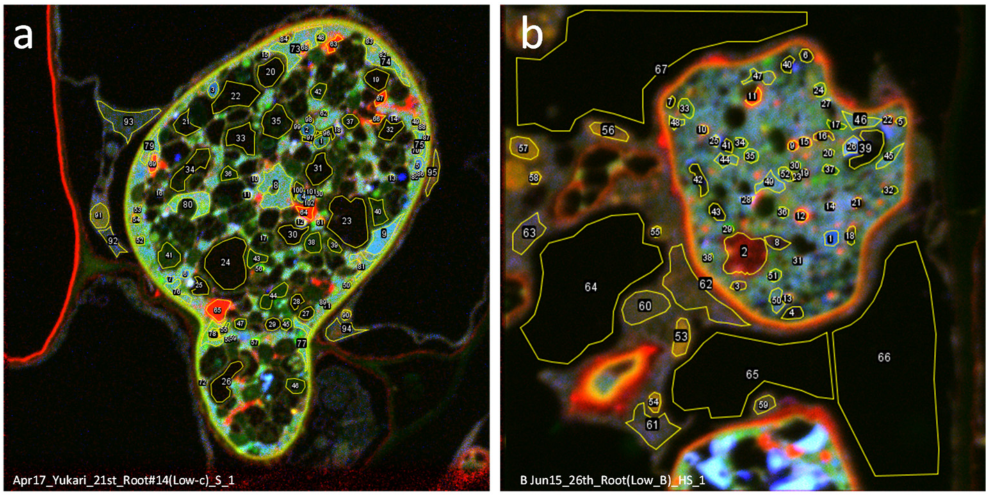

Figure S2. Examples of ROIs of Figure 2.

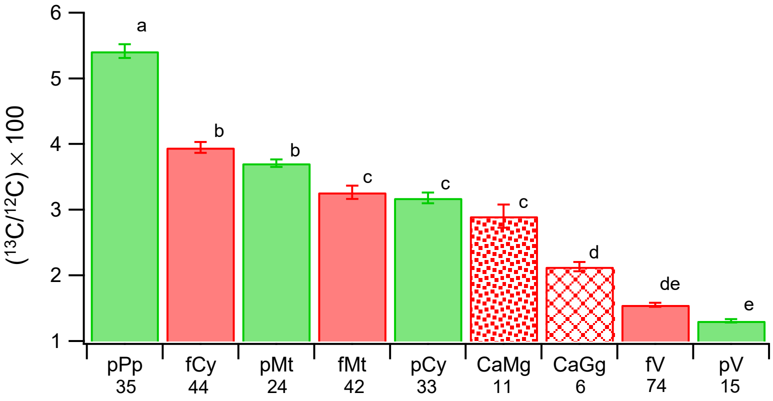

Figure S3. Multiple comparisons of r13C of all structures (all ROIs of gram-negative and positive bacteria, and fungal and plant organelles).

CaGg, *Candidatus* Glomeribacter gigasporarum; CaMg, *Candidatus* Moeniiplasma glomeromycotorum; fCy, fungal cytoplasm; fMt, fungal mitochondrion; fV, fungal vacuole; pCy, plant cytoplasm; pMt, plant mitochondrion; pPp, plant plastid; pV, plant vacuole. Different letters show statistically significant differences among structures based on the Tukey–Kramer method at $p < 0.05$. Bar, standard error of the mean. (N = number of ROI, shown under each structure).

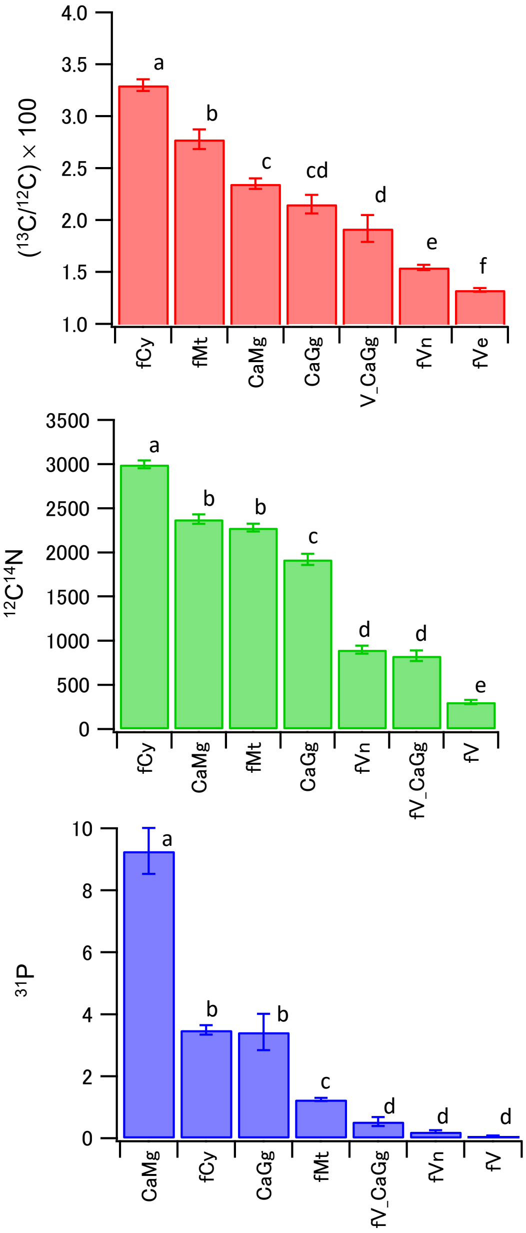

Figure S4. ROI analysis of fungal structures of image #4 (Figure S2. a). r13C (red), $^{12}C^{14}N$ (green), ^{31}P (blue). CaGg, *Candidatus* Glomeribacter gigasporarum; CaMg, *Candidatus* Moeniiplasma glomeromycotorum; fCy, fungal cytoplasm; fMt, fungal mitochondrion; fV, fungal vacuole; fVn, N-rich fungal vacuole; V_CaGg, *Ca*Gg residing fungal vacuole. Tukey-Kramer method. Different letters show statistically significant differences among structures based on the Tukey–Kramer method at $p < 0.05$. Bar, standard error of the mean. (N = number of ROI, shown under each structure).

Table S1. Averages of r13C, ^{31}P, and $^{12}C^{14}N$ of ROIs of seven images analyzed.

Image		CaMg/CaGg	ROI: Structures								
			CaGg	CaMg	fMt	fCy	fV	pPp	pMt	pCy	pV
#2	ROI#		n.d.	2	7	7	10	7	5	5	4
	r13C		n.d.	3.22	3.34	4.71	1.82	5.43	3.97	2.97	1.38
	^{31}P		n.d.	20.95	4.38	11.86	1.61	1.84	4.38	4.36	0.26
	$^{12}C^{14}N$		n.d.	4420.31	4211.25	6277.85	1192.96	3905.06	3038.78	2447.66	585.60
#3	ROI#		1	2	9	8	10	n.d.	n.d.	3	n.d.
	r13C	Δ1.96	1.99	2.94	3.66	4.29	1.55	n.d.	n.d.	3.35	n.d.
	^{31}P	2.64	11.96	31.56	7.36	19.72	4.26	n.d.	n.d.	7.96	n.d.
	$^{12}C^{14}N$	1.23	4119.15	5077.14	4549.00	6518.98	989.10	n.d.	n.d.	2024.18	n.d.
#4 (Figure S2. a)	ROI#		4	3	8	10	17	2	n.d.	4	n.d.
	r13C	Δ1.17	2.15	2.35	2.78	3.30	1.33	3.44	n.d.	2.31	n.d.
	^{31}P	2.70	3.43	9.27	1.25	3.50	0.08	0.70	n.d.	1.33	n.d.
	$^{12}C^{14}N$	1.24	1922.09	2375.88	2280.57	2996.86	305.30	1974.16	n.d.	1139.51	n.d.
#13 (Figure S2. b)	ROI#		n.d.	1	12	7	5	6	2	3	4
	r13C		n.d.	2.65	3.34	3.85	1.86	5.26	3.44	3.22	1.19
	^{31}P		n.d.	30.13	8.50	16.54	3.49	3.83	6.79	7.34	0.12
	$^{12}C^{14}N$		n.d.	3817.71	4492.25	4823.74	1203.48	3684.64	2524.94	2278.70	402.99
#14	ROI#		n.d.	1	n.d.	1	13	10	9	8	n.d.
	r13C		n.d.	3.10	n.d.	3.83	1.38	5.50	3.61	3.45	n.d.
	^{31}P		n.d.	34.26	n.d.	6.81	0.71	2.23	5.37	7.72	n.d.
	$^{12}C^{14}N$		n.d.	5054.76	n.d.	6572.26	1051.36	4147.33	2900.28	2769.79	n.d.
#15	ROI#		1	1	3	6	9	n.d.	n.d.	n.d.	n.d.
	r13C	Δ1.55	2.10	2.70	2.70	3.67	1.74	n.d.	n.d.	n.d.	n.d.
	^{31}P	2.40	13.19	31.60	7.39	16.50	1.33	n.d.	n.d.	n.d.	n.d.
	$^{12}C^{14}N$	1.02	4280.44	4365.54	4230.73	5993.89	1073.89	n.d.	n.d.	n.d.	n.d.
#21	ROI#		n.d.	1	3	5	10	10	8	10	7
	r13C		n.d.	4.15	3.39	4.10	1.53	5.79	3.73	3.34	1.33
	^{31}P		n.d.	15.56	4.38	8.22	1.09	2.40	4.43	5.38	0.17
	$^{12}C^{14}N$		n.d.	3781.67	3322.95	3753.37	853.69	2993.93	2210.50	2007.32	492.97

- r13C: $(^{13}C/^{12}C)*100$
- ΔCaMg/CaGg = (CaMg-1)/(CaGg-1)
- CaGg, *Candidatus* Glomeribacter gigasporarum; CaMg, *Candidatus* Moeniiplasma glomeromycotorum; fMt, fungal mitochondrion; fCy, fungal cytoplasm; fV, fungal vacuole; pPp, plant pastid; pMt, plant mitochondrion; pCy, plant cytoplasm; pV, plant vacuole.
- n.d.: the organelles or cells are not exist or analyzed.
- Values of $^{12}C^{14}N$ and ^{31}P were normalized by a counting time.

Table S2. r13C of structures and the multiple comparison within plant and fungus (Tukey–Kramer method)

		# of Sample	r13C Mean	Unbiased variance	Standard deviation	Standard error of the mean
Plant	pPp	5	5.09 [a]	0.89	0.94	0.42
	pMt	4	3.69 [b]	0.05	0.22	0.11
	pCy	6	3.11 [b]	0.18	0.42	0.17
	pV	3	1.30 [c]	0.01	0.10	0.06
Fungus	fCy	7	3.96 [a]	0.21	0.45	0.17
	fMt	6	3.20 [b]	0.14	0.38	0.15
	CaMg	7	3.02 [b]	0.34	0.58	0.22
	CaGg	3	2.08 [c]	0.01	0.08	0.05
	fV	7	1.60 [c]	0.05	0.21	0.08

- r13C: ($^{13}C/^{12}C$)*100
- CaGg, *Candidatus* Glomeribacter gigasporarum; CaMg, *Candidatus* Moeniiplasma glomeromycotorum; fMt, fungal mitochondrion; fCy, fungal cytoplasm; fV, fungal vacuole; pPp, plant pastid; pMt, plant mitochondrion; pCy, plant cytoplasm; pV, plant vacuole.
